# The Interplay between *Entamoeba* and Enteropathogenic Bacteria Modulates Epithelial Cell Damage

**DOI:** 10.1371/journal.pntd.0000266

**Published:** 2008-07-23

**Authors:** José Manuel Galván-Moroyoqui, M. del Carmen Domínguez-Robles, Elizabeth Franco, Isaura Meza

**Affiliations:** Departamento de Biomedicina Molecular, Centro de Investigación y de Estudios Avanzados del IPN, México DF, México; New York University School of Medicine, United States of America

## Abstract

**Background:**

Mixed intestinal infections with *Entamoeba histolytica*, *Entamoeba dispar* and bacteria with exacerbated manifestations of disease are common in regions where amoebiasis is endemic. However, amoeba–bacteria interactions remain largely unexamined.

**Methodology:**

Trophozoites of *E. histolytica* and *E. dispar* were co-cultured with enteropathogenic bacteria strains *Escherichia coli* (EPEC), *Shigella dysenteriae* and a commensal *Escherichia coli.* Amoebae that phagocytosed bacteria were tested for a cytopathic effect on epithelial cell monolayers. Cysteine proteinase activity, adhesion and cell surface concentration of Gal/GalNAc lectin were analyzed in amoebae showing increased virulence. Structural and functional changes and induction of IL-8 expression were determined in epithelial cells before and after exposure to bacteria. Chemotaxis of amoebae and neutrophils to human IL-8 and conditioned culture media from epithelial cells exposed to bacteria was quantified.

**Principal Findings:**

*E. histolytica* digested phagocytosed bacteria, although *S. dysenteriae* retained 70% viability after ingestion. Phagocytosis of pathogenic bacteria augmented the cytopathic effect of *E. histolytica* and increased expression of Gal/GalNAc lectin on the amoebic surface and increased cysteine proteinase activity. *E. dispar* remained avirulent. Adhesion of amoebae and damage to cells exposed to bacteria were increased. Additional increases were observed if amoebae had phagocytosed bacteria. Co-culture of epithelial cells with enteropathogenic bacteria disrupted monolayer permeability and induced expression of IL-8. Media from these co-cultures and human recombinant IL-8 were similarly chemotactic for neutrophils and *E. histolytica.*

**Conclusions:**

Epithelial monolayers exposed to enteropathogenic bacteria become more susceptible to *E. histolytica* damage. At the same time, phagocytosis of pathogenic bacteria by amoebae further increased epithelial cell damage.

**Significance:**

The *in vitro* system presented here provides evidence that the *Entamoeba*/enteropathogenic bacteria interplay modulates epithelial cell responses to the pathogens. In mixed intestinal infections, where such interactions are possible, they could influence the outcome of disease. The results offer insights to continue research on this phenomenon.

## Author Summary

In amoebiasis, a human disease that is a serious health problem in many developing countries, efforts have been made to identify responsible factors for the tissue damage inflicted by the parasite *Entamoeba histolytica.* This amoeba lives in the lumen of the colon without causing damage to the intestinal mucosa, but under unknown circumstances becomes invasive, destroying the intestinal tissue. Bacteria in the intestinal flora have been proposed as inducers of higher amoebic virulence, but the causes or mechanisms responsible for the induction are still undetermined. Mixed intestinal infections with *Entamoeba histolytica* and enteropathogenic bacteria, showing exacerbated manifestations of disease, are common in endemic countries. We implemented an experimental system to study amoebic virulence in the presence of pathogenic bacteria and its consequences on epithelial cells. Results showed that amoebae that ingested enteropathogenic bacteria became more virulent, causing more damage to epithelial cells. Bacteria induced release of inflammatory proteins by the epithelial cells that attracted amoebae, facilitating amoebic contact to the epithelial cells and higher damage. Our results, although a first approach to this complex problem, provide insights into amoebic infections, as interplay with other pathogens apparently influences the intestinal environment, the behavior of cells involved and the manifestations of the disease.

## Introduction

Once trophozoites of *Entamoeba histolytica* reach the host intestine, they can damage the mucosa epithelial layer and spread through the submucosa and the lamina propia and other tissues. Neutrophils and other cells infiltrate the tissue in the vicinity of amoebic lesions increasing the inflammatory response and tissue damage [1],[2]. In contrast, *Entamoeba dispar*, an amoeba that colonizes the human intestine together with *E. histolytica* and that is morphologically indistinguishable and genetically very similar to the latter, is not invasive and does not produce the clinical manifestations of an *E. histolytica* intestinal infection [3],[4],[5].

Search for expression of genes that could be correlated with the difference in pathogenicity between *E. histolytica* and *E. dispar* has mainly revealed higher expression in the former, of molecules involved in lysis of target cells , such as the amoebapore and specific cysteine proteinases [6],[7],[8]. Nonetheless, *E. histolytica* trophozoites can also remain as commensals in the intestinal lumen without causing manifestations of disease [9],[10].

It has been proposed that *E. histolytica* pathogenicity could be induced by ingestion of bacteria present in the host intestine. *In vitro* experiments have shown that after phagocytosis of an *E. coli* non-pathogenic laboratory strain (*Ec*346), trophozoites of *E. histolytica* increased their virulence together with their adhesive properties to target cells [11],[12],[13]. However, the same authors reported that long time cultivation with this bacteria strain rendered the amoebae less virulent [13]. In spite of the possible important role of intestinal bacteria in amoebic behavior in their natural habitat, little has been explored or elucidated about responses triggered by bacteria/amoeba interplay that could be important in the induction of tissue invasion and disease.

Pro-inflammatory cytokines released by cultured epithelial and endothelial cells after viral or bacterial infections induce structural and functional alterations in non-infected cells. These alterations lead to increased monolayer permeability and disarray of intercellular junctions and the cortical cytoskeleton which would facilitate passage of pathogens [14],[15],[16],[17]. Experimental infection of intestinal animal models with *E. histolytica* trophozoites has shown that pro-inflammatory cytokines released by epithelial cells are activated by amoebic cysteine proteinases. It has been proposed that activated cytokines would then recruit neutrophils and other inflammatory cells to the sites of infection, suggesting an important role of the host inflammatory response in tissue damage [17],[18],[19],[20].

In regions where amoebiasis is endemic mixed intestinal infections with *E. histolytica*/enteropathogenic bacteria are common [21],[22],[23],[24],[25],[26]. It is also well established that co-infection with the non-pathogenic *E. dispar* is prevalent in these regions [9],[10],[27]. How amoeba/bacteria interplay in these infections could modify disease manifestations by modulating pathogen virulence and the host response, has not been determined.

We approached this problem analyzing the interaction of *E. histolytica and E. dispar* with two pathogenic enterobacteria strains frequently associated with mixed infections, EPEC and *Shigella dysenteriae*, isolated from infected individuals. The results were compared with those obtained with amoebae that were not interacted with bacteria and with amoebae that phagocytosed a commensal *E. coli* strain. The response of epithelial cells to bacteria exposure and the effects of this exposure on cell damage by amoebae were then investigated. *E. histolytica*/enteropathogenic bacteria interactions induced higher virulence of amoebae. Enteropathogenic bacteria altered the epithelial barrier and induced release of chemoattractant molecules for both neutrophils and *E. histolytica* and better adhesion of amoebae to epithelial cells with subsequent increases of the cytopathic effect. *In vivo*, one could hypothesize that such conditions might confer higher susceptibility to pathogen invasion and severe disease manifestations. Our observations about survival and escape of infectious bacteria from amoebae, although preliminary, could be an interesting factor to consider when studying the intestinal environment of mixed infections.

## Materials and Methods

### Cells

*E. histolytica* HM1-IMSS trophozoites were cultured in TYI-S-33 medium as indicated [28] after their recovery from hamster liver passage and determination of virulence by production of liver abscesses. *E. dispar* SAW 760 RR, clone 2, trophozoites were cultured in axenic medium LY-S-2 as reported [29]. The bacteria utilized corresponded to clinical isolates of the commensal *Escherichia coli* 086:H18 and the pathogenic EPEC B171-0111: NM, kindly donated by Dr. Teresa Estrada (CINVESTAV, México) and *Shigella dysenteriae* kindly provided by Dr. Celia Alpuche (Pediatrics Hospital, National Institutes of Health, Mexico).

MDCK (NBL-2), dog kidney epithelial cells, passage 72, were grown to form confluent polarized monolayers as previously reported [30]. Cells were seeded on 24-well culture dishes for interaction with amoebae or bacteria, or on Millicel filters for transepithelial resistance measurements. Monolayers grown on glass cover slips were used for fluorescence microscopy observations.

### Phagocytosis and viability of bacteria

To standardize the conditions to measure phagocytosis of bacteria by amoebae and bacteria viability inside amoebae, bacteria were transfected with vector pd2EGFP (Clontech Laboratories, Palo Alto, CA) to express green fluorescent protein, as reported [31]. EGFP-expressing bacteria were co-cultured with amoebae in amoeba/bacteria ratios of 1∶100 for different periods of time. Amoebae were then freed of non-phagocytosed bacteria by extensively rinsing the culture wells with PBS containing 5 mM sodium azide and 50 µM gentamycin. Attached amoebae were gently detached with PBS-gentamycin solution and residual extracellular bacteria removed by centrifugation-resuspension cycles in the same solution. Aliquots of the pelleted amoebae were further checked by fluorescence microscopy for absence of bacteria. Pellets were then fixed with 3.7 % formaldehyde, washed with PBS and resuspended in 300 µl of PBS. Intracellular EGFP-fluorescence was determined by flow cytometry. The highest fluorescence values corresponding to the highest number of bacteria ingested by amoebae were registered at 2 and 3 h after the interaction.We chose two and a half hours as the optimal time for phagocytosis. Amoebae that phagocytosed bacteria (not expressing EGFP) were utilized for all the following experiments. Cell cultures were freed of bacteria following the techniques applied to fluorescent bacteria and proven to be effective for this purpose. For the viability assays, bacteria expressing EGFP were interacted with amoebae in the above conditions. The capacity of bacteria, recovered from amoebae at different times after been phagocytosed, to form colonies on LB-agar plates was measured by CFU assays. At the indicated times, amoeba cultures were freed of non-phagocyted bacteria as described above. Amoebae were lysed with 0.12% Triton X-100 in LB medium and 100 µl of serial dilutions (up to 10^−4^) of the lysate added to LB agar plates and incubated at 37°C. Colonies formed in each plate after 24 h were quantified. One hundred per cent viability corresponds to the number of bacteria forming colonies 2.5 h after been phagocytosed by amoebae. Quantification of colonies formed by bacteria strains not expressing EGFP and recovered from amoebae in the above conditions revealed similar viability to that registered for EGFP-expressing bacteria.

### Cytopathic effect

*E. histolytica* trophozoites (2×10^5^) were co-cultured with bacteria strains not expressing EGFP for 2.5 h in amoeba/bacteria ratios of 1∶100. After removal of non-phagocytosed bacteria, as indicated above, amoebae were deposited on confluent MDCK cell monolayers. After one hour of co-cultivation at 37°C, amoebae were removed by keeping the culture dishes in ice for 5 min and extensive rinsing with ice-cold PBS. The remaining intact epithelial cells were quantified by staining with the methylene blue method [32]. The number of intact cells in MDCK cell monolayers not exposed to amoebae was the control for 0 % cytopathic effect. Inhibition by 100 mM galactose or 250 µM E-64 was measured in amoebae incubated for 30 min before their addition to the epithelial monolayers.

### Adhesion of amoebae to MDCK cells

*E. histolytica* trophozoites were labeled with 1.0 µl of calcein AM (Molecular Probes, Eugene OR) incubating at 37°C for 30 min. After washing with PBS, amoebae were checked for viability with trypan blue and co-cultured with each of the bacteria strains for 2.5 h. Non-phagocytosed bacteria were removed as described above and amoebae tested for adhesion to formaldehyde-fixed MDCK confluent monolayers for 20 min, as reported [13]. Adhered amoebae were detached by rinsing with ice-cold PBS, fixed with 3.7 % formaldehyde and their fluorescence measured at 517 nm by flow cytometry. Adhesion indexes of calcein-labeled amoebae to MDCK monolayers, previously exposed to bacteria for 4 h, as well as those of amoebae pretreated with 100 mM galactose or the Gal/GalNac lectin polyclonal antibody (10 µg/10,000 amoebae) were estimated in the same way.

### Fluorescence measurement of Gal/GalNac lectin on the surface of amoebae

After interaction of bacteria as indicated above, trophozoites were fixed with 2% paraformaldehyde for 20 min and rinsed 3X with PBS. A polyclonal antibody to the Gal/GalNac 170 kDa subunit (H5), kindly donated by Dr. Barbara Mann, was added at 1∶50 dilution in PBS/2% FBS to a suspension of 2.5×10^5^ trophozoites and these incubated at 4°C for 40 min. A secondary antibody (Goat anti-Rabbit IgG tagged with FITC) was added to the cells at a dilution of 1∶400 and incubated for 45 min at 4°C. Cells were rinsed with PBS and resuspended in 300 µl of PBS/2% paraformaldehyde. FITC fluorescence was measured in a FACSCalibur flow cytometer at emission peak of 520 nm.

### Cysteine proteinase activity

Ezymatic activity of cysteine proteinases (CP) in *E. histolytica* lysates and in culture medium was analyzed in control amoebae and amoebae co-cultured for 2.5 h with the bacteria strains. After removing non-phagocytosed bacteria as indicated above, trophozoites were cultured for 2 h in culture medium without serum and lysed by freeze-thaw cycles in 50 mM Tris-HCl, pH 7.2, 150 mM NaCl, 1.0 mM CaCl_2_. Two micrograms of each of the trophozoite lysates and 5 µl of their respective culture medium (freed of debris by centrifugation at 15,000 xg) were loaded in 1% gelatin, 10% polyacrylamide gels using reported conditions to analyze individual CP activities [32]. The clear areas in the gels revealed cysteine proteinase activity by digestion of the gelatin. Enzymatic activity areas were scanned with the SigmaGel Program in gel from three independent experiments. Lysates and culture media were also separated by electrophoresis in Laemmli's 10% SDS-polyacrylamide gels and silver-stained as controls for protein loading.

### Transepithelia resistence (TER) measurement

MDCK cells were plated on polycarbonate filters (1.2 cm diameter, Millipore Co, Bedford, MA) previously coated with a solution containing 30 mg/ml of rat Type I collagen. After cells reached confluence, approximately after 48 h in culture, bacteria were added in a ratio of 100∶1. TER was registered as described [30], before adding bacteria and at different times of co-culture, previous removal of bacteria and addition of fresh culture medium. FITC-labeled annexin V, Rhodamine-phalloidin and DAPI (Molecular Probes, Eugene, OR) were utilized to stain cells and monitor apoptosis, cell morphology and organization of the cytoskeleton in MDCK monolayers exposed to bacteria. For this, cells grown on cover slips were fixed with 3.7 % paraformaldehyde and stained by standard fluorescence microscopy methods recommended for these indicators. A monolayer irradiated with UV light was the positive control for apoptosis

### Chemotaxis to pro-inflammatory cytokine, IL-8 and conditioned culture media

Chemotaxis was assayed in Transwell chambers as previously reported [33], loading 150,000 calcein-labeled amoebae in the upper chambers resuspended in migration buffer (50 mM Tris-HCl pH 7.2, 150 mM NaCl, 1 mM Ca Cl_2_, 0.01% BSA). Amoebae that had not ingested bacteria as well as amoebae that phagocytosed bacteria for 2.5 h were tested. The lower chambers contained solutions containing 100 ng/ml in migration buffer of recombinant human cytokine IL-8 (Preprotech Inc., Rocky Hill, NJ), conditioned culture media obtained from MDCK monolayers co-cultured with each of the bacteria strains in the absence of serum or media from overnight bacteria cultures. Bacteria were removed by centrifugation before adding the culture media to the lower chambers. The number of calcein-labeled amoebae that migrated to the lower chambers was determined by flow cytometry. Culture media from monolayers not exposed to bacteria were used as control. Chemotactic index (CI) was calculated considering CI = % chemotactic migration/ % random migration. CI = 1.0 corresponds to migration to control media. Purified canine neutrophils were obtained from citrate-treated dog blood, separated in 20 % Ficoll gradients. The layer containing neutrophils was further purified by resuspension/centrifugation cycles in PBS and labeled with calcein. Eighty thousand cells were loaded in the upper chambers and chemotaxis assessed as indicated for amoebae.

### RT-PCR assays

Total RNA was obtained from control MDCK monolayers, monolayers exposed to *E. coli* strains, exposed to *S. dysenteriae* or from monolayers incubated with 10 µM of BAY11-7085 (inhibitor of NFκB activation, Calbiochem, La Jolla, CA), previous exposure to *Shigella*. RNA was extracted with TRIzol (Invitrogen, Rockville, MD) following the specifications of the manufacturer. cDNA was synthesized from 5 µg of DNAse I-treated RNA (DNA-free™, Ambion Inc.) in a reaction mixture containing 5 mM Mg Cl_2_, 50 mM KCl, 10 mM Tris-HCl, pH 8.3, 0.25 mM of each dNTP, 40U of RNAse inhibitor, 0.5 µM of oligo-dT-primers and 50U of Superscript II (Invitrogen). The reactions were allowed to proceed for 45 min at 42°C and inactivated for 5 min at 65°C. Amplification of IL-8 cDNA was done by mixing 1 µl of cDNA with 50 µl of PCR buffer supplemented with 2.5 mM MgCl_2_, 0.5 µM each of sense (5′ATGACTTCCAAGCTGGCTG3′) and antisense (5′TCTGAGTTTTCACAATGTGG3′) primers (designed accordingly to the IL-8 mRNA canine sequence, *Canis familiaris*) and 1U of Taq-polymerase (Invitrogen). PCR cycle conditions were 30 s at 94°C, 20 s at 45°C and 1 min at 72°C for 32 cycles. Sense (5′ATGGATGATGATATCGCCGC3′) and antisense (5′TTGGGGTTCAGGGGGGC3′) primers were utilized for amplification of the canine β-actin cDNA. The resulting RT-PCR products were analyzed in 1% agarose gels stained with a 2 µg/ml solution of ethidium bromide to monitor the presence of the expected size bands corresponding to IL-8 (194 pb) and β-actin (338 bp).

### Statistics

Data are presented as means±standard deviation. The significance of the results was calculated by *t*–Student test utilizing the program Sigma Stat. *p values≤0.05 were considered significant respect to controls. n values correspond to at least 3 independent experiments done in duplicate.

### Materials

Unless otherwise specified, all reagents were obtained from Sigma Chemical (St. Louis, MO).

## Results

### Cytopathic effect of amoebae after phagocytosis of bacteria

**Figure 1:**
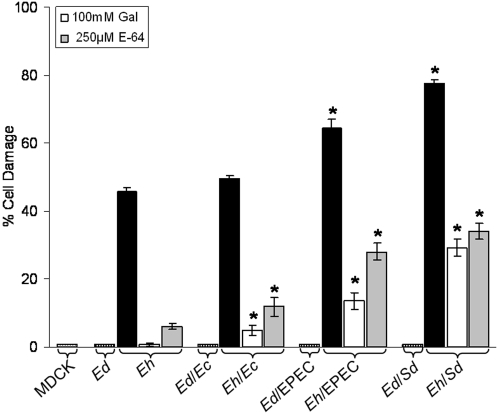
Cytopathic effect of amoebae after phagocytosis of bacteria. Amoebae and bacteria (1∶100) were co-cultured for 2.5 h. After removal of non-phagocyted bacteria, amoebae were co-cultured with confluent MDCK cell monolayers for 1 h. Cytopathic effect is expressed as percent cell damage relative to 0% damage calculated from the number of intact cells in monolayers that were not exposed to amoebae (MDCK). *Eh*, *Ed* bars represent values obtained for monolayers exposed to *E. histolytica* or *E dispar* that did not phagocytose bacteria. *Ec* corresponds to commensal *E. coli* 086∶H18, EPEC to enteropathogenic *E. coli* and *Sd* to *Shigella dysenteriae*. 100 mM galactose or 250 µM E-64 were utilized to compete or inhibit the cytopathic effect. Bars represent mean±SD, n = 9, * *p*≤0.05.

After a 2.5 h incubation of *E. histolytica* and *E. dispar* trophozoites with *E. coli* 086∶H18 (*Ec*), EPEC, or *Shigella dysenteriae* (*Ed*), the cytopathic effect of amoebae on MDCK cell monolayers was quantified and expressed as percentage of cell damage (Figure 1). Amoebae that were not incubated with bacteria (*Eh* or *Ed*) were controls for damage inflicted by amoebae that phagocytosed bacteria. Phagocytosis of *E. coli* 086∶H18 by *E. histolytica* (*Eh/Ec*) increased its cytopathic effect, but the increase was not significantly higher than that caused by control amoebae (49.6±0.95 versus 46.0±1.08). However, after phagocytosis of EPEC (*Eh*/EPEC) or *Shigella* (*Eh*/*Sd*), the cytopathic effect of *E. histolytica* increased to 64.6±2.63 and 77.6±1.24, respectively. In contrast to what was observed with *E. histolytica*, phagocytosis of any of the bacterial strains by *E. dispar* did not induce cytopathic behavior (*Ed/Ec, Ed*/EPEC, *Ed/Sd*). Figure 1 also shows that 100 mM Galactose, a known ligand of the amoebic surface Gal/GalNAc lectin, drastically reduced the cytopathic effect of control amoebae to less than 1.0 %. The same concentration of the sugar inhibited cell damage 5 %, 13 % and 29 % in amoebae that phagocytosed the commensal *E. coli*, EPEC or *Shigella*, respectively. Furthermore, incubation of amoebae with E-64, a specific inhibitor of cysteine proteinase activity at concentrations not deleterious to amoebic viability [7],[32], also caused significant inhibition of the cytopathic effect: 82 % in control amoebae, 76 %, 66 % and 55 % in *E. coli*, EPEC and *Shigella*, respect to their cytopathic effect in absence of E-64.

These results showed that phagocytosis of bacteria did not induce virulence in *E. dispar*, while in *E. histolytica* it induced an increase of the cytopathic effect particularly after phagocytosis of pathogenic bacteria. Galactose and E-64 inhibition suggest a role for the lectin and cysteine proteinase in the induction of enhanced virulence. As *E. dispar* did not show induction of virulence in any of the conditions tested, the following results refer only to *E. histolytica*.

### Surface expression of the Gal/GalNAc lectin in amoebae and their adhesion after phagocytosis of bacteria

**Figure 2:**
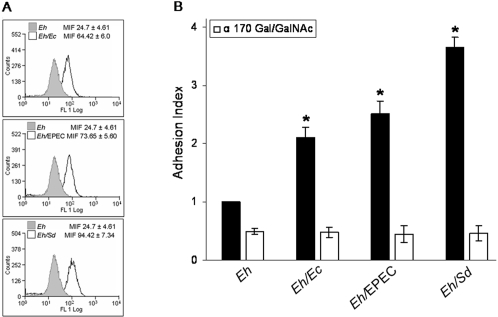
Adhesion and surface expression of the Gal/GalNAc lectin in amoebae after phagocytosis of bacteria. A, Representative histogram of MIF of the Gal/GalNac lectin on the surface of 10,000 amoebae before (*Eh*) and after phagocytosis of *E.coli* (*Eh/Ec*), EPEC (*Eh/*EPEC) and *Shigella* (*Eh/Sd*). The numbers above the histograms represent mean±SD, n = 3, *p*≤0.05. B, calcein-labeled amoebae were co-cultured with bacteria for 2 h and after removal of non-phagocyted bacteria, co-cultured with MDCK formaldehyde-fixed monolayers for 20 min. Adhered amoebae were detached and counted by flow cytometry. Adhesion index was calculated taking as value 1.0 the adhesion of control amoebae. Bars represent mean±SD, n = 6, * *p*≤0.05.

Since amoebic adhesion to target cells depends on the activity of the Gal/GalNAc lectin, we analyzed its concentration in *E. histolytica* trophozoites before and after phagocytosis of bacteria. Amoebae were fixed and labeled with a polyclonal antibody directed to the 170 kDa heavy subunit of the lectin that contains the galactose-binding domain, and a secondary antibody tagged with FITC. Fluorescence intensity on the surface was measured in 10,000 cells for each condition. Figure 2A shows the mean index of fluorescence (MIF) in a representative experiment. Amoebae that were not exposed to bacteria (*Eh*) showed a MIF = 24.7±4.61. Amoebae that phagocytosed *E. coli* (*Eh/Ec*) showed a MIF = 64.42±6.0. Amoebae that phagocytosed EPEC (*Eh/*EPEC) showed a MIF = 73.65±5.60 and those that phagocytosed *Shigella* (*Eh/*S*d*) showed a MIF = 94.42±7.34. The average of MIF values, obtained in three independent experiments, indicated 3.0-fold and 3.9-fold increases in amoebae after phagocytosis of EPEC or *Shigella.* Phagocytosis of the commensal *E. coli* only induced a 2.0-fold increase. Treating amoebae with only the secondary antibody did not increased MIF values.

Augmented Gal/GalNAc lectin concentration on the surface of amoebae could result in better adhesion and higher cytopathic effect, thereby adhesion of amoebae that phagocytosed bacteria to MDCK cells was quantified (Figure 2B). The adhesion index for control amoebae (not incubated with bacteria, *Eh*) was given value 1.00. After phagocytosis of the commensal *E. coli*, the adhesion index of trophozoites was 2.17±0.12. After phagocytosis of EPEC, the adhesion index was further increased to 2.52±0.21 and for amoebae that phagocytosed *Shigella* it reached values of 3.65±0.18. The specificity of the adhesion was corroborated in assays where amoebae (control as well as those that phagocytosed bacteria) were preincubated with the polyclonal antibody to the amoebic Gal/GalNAc lectin before interaction with the cells. The competition with the antibody reduced adhesion indexes in all the cases to 50% of the control value. An irrelevant antibody of the same isotype did not compete the adhesion of amoebae.

These results showed that phagocytosis of bacteria, but particularly pathogenic bacteria, induced a higher concentration of the Gal/GalNAc lectin on the surface of amoebae that seems correlated with a lectin-mediated increase of amoebic adhesion to MDCK cells. However, the antibody could only decrease binding in all the cases to the same level, suggesting that increased adhesion of amoebae after phagocytosis of bacteria is mainly, but not completely lectin-dependent.

### Cysteine proteinase activitiy in amoebae after phagocytosis of bacteria

**Figure 3:**
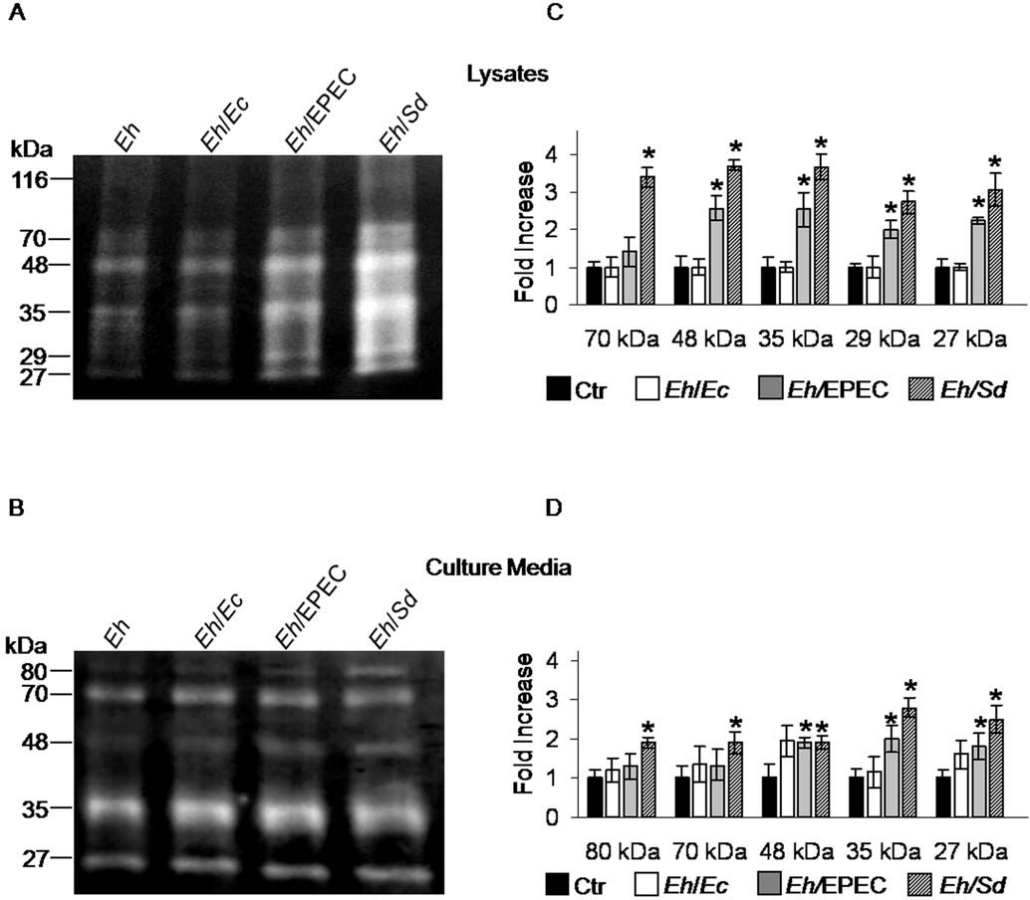
Cysteine proteinase activities in amoebae after phagocytosis of bacteria. A, a representative gelatin zymogram of amoebic lysates after phagocytosis of bacteria. B, representative zymogram of the culture media obtained from the same amoebae utilized in Figure 3A. The bars next to the gels (C and D) show the values obtained after densitometric scanning of each band. Values correspond to fold increase over value 1.0 given to each band in lysates and culture media of amoebae that did not phagocytose bacteria (*Eh*). Mean±SD, n = 3, * *p*≤0.05.

The results in Figure 1 showing that the increase in cytopathic effect of amoebae that phagocytosed bacteria could be inhibited by E-64, led us to analyze cysteine proteinase (CP) activitiy in these amoebae. Figure 3 shows representative gelatin zymograms of lysates and culture media of *E. histolytica* trophozoites after phagocytosis of bacteria. The enzymatic activity of each proteinase, corresponding to the area of gelatin digested, was quantified by densitometry (Figure 3A and 3B). The bars in Figure 3C and 3D, express the fold-increase over value 1.0, given to each of the digested areas in lysates and culture media from control amoebae not exposed to bacteria (*Eh*). Proteinase activity bands corresponding to 48, 35, 29 and 27 kDa have been identified in lysates of trophozoites as the major proteinases CP1, CP2, and CP5 [6]. Figure 3C shows the values obtained from 3 separate gels where the enzymatic activities of CP1 and CP2 increased above two and three-fold in lysates of amoebae that phagocytosed EPEC or *Shigella*. The bands of 29 and 27 kDa corresponding to CP5 showed a lower but significant increase above control amoebae that however, was not significant between EPEC and Shigella for the 29 kDa band. After phagocytosis of the commensal *E. coli* the increase in proteinase activities was not significant. The highest fold-increase for all the activities was observed after phagocytosis of *Shigella.* The enzymatic activity band of 70 kDa, present in both zymograms, may represent an aggregate of some of the major proteinase activities, as it does not correspond to characerized proteinases.

Cysteine proteinases released to the medium of amoebae cultured in axenic conditions have been identified as CP1, CP3 and CP5 [6]. Zymograms of culture media without serum in which the amoebae were kept for 2 hours after phagocytosis of bacteria (Figure 3B), showed an increase close to 2-fold or higher for all the bands in *Shigella.* For EPEC the increase was lower, but still significant for bands of 48 kDa, 35 kDa and 27 kDa (Figure 3D). Parallel 10% SDS-polyacrylamide gels of lysates and culture media were silver stained to visualize all the protein bands in the gels (Figure S2). The gels show that the same protein concentration was loaded in all lanes and no particular difference was observed in particular bands. Therefore, the differences in enzymatic activity in the zymograms are real, indicating specific activation of some proteinases in the amoebae that phagocytosed bacteria. The observation that E-64 substantially inhibited the increase in cytopathic effect induced by phagocytosis of bacteria, shown in Figure 1, supports that these enzymes could have an important role in the induction of higher virulence.

### MDCK monolayers exposed to bacteria are more susceptible to amoebic virulence

**Figure 4:**
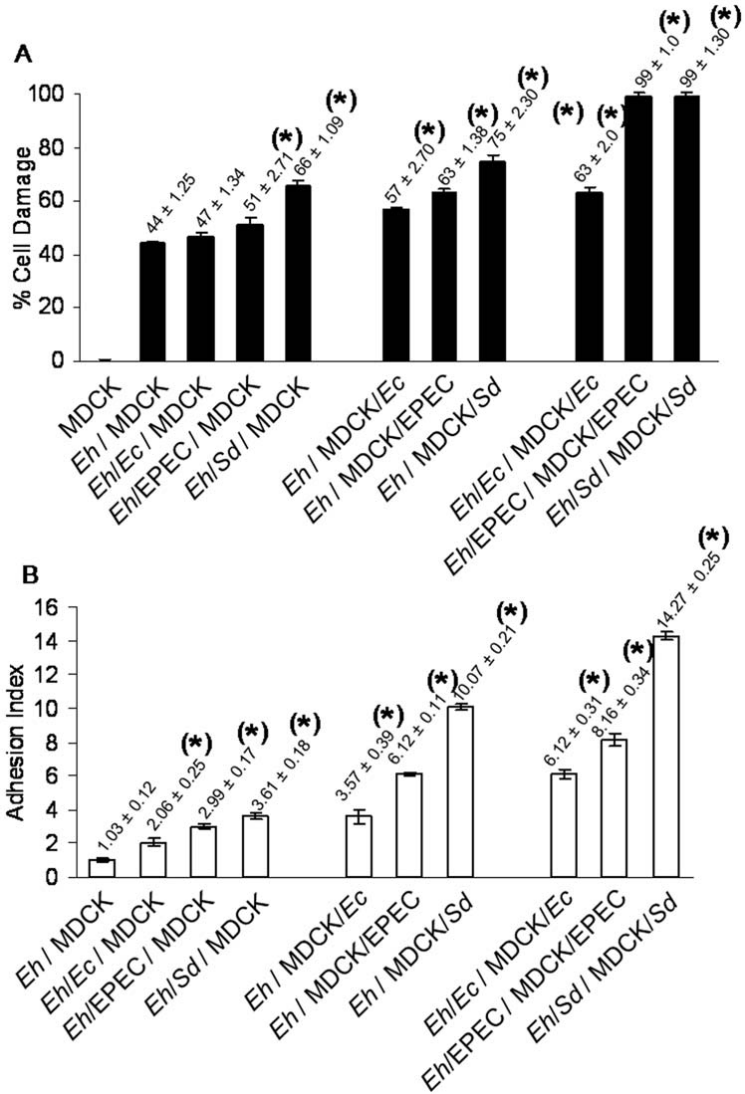
MDCK monolayers exposed to bacteria are more susceptible to amoebic virulence. A, Cell Damage. The first set of bars from the left shows that amoebae that phagocytosed pathogenic bacteria induced higher damage to MDCK regular monolayers. The second set of bars shows that amoebae that had not phagocytosed bacteria can also cause higher damage if the MDCK monolayers had been exposed to bacteria. The third set of bars shows that damage to the monolayers reached the highest values, if both, amoebae and epithelial cells had been incubated with bacteria. B, Adhesion Index. The first set of bars from the left shows that amoebae that phagocytosed bacteria, particularly pathogenic bacteria, increased their adhesion index to MDCK monolayers. The second set indicates that adhesion of amoebae can also be increased if MDCK monolayer had been exposed to bacteria. The third set shows an even higher increase of adhesion if amoebae and epithelial cells had been exposed to bacteria. Numbers above the bars indicate mean±SD, n = 6, * *p*≤0.01.

From the results above, it is possible to think that in mixed amoeba/bacteria infections, the interplay of pathogens might modulate damage to the epithelial cells, up-regulating expression of specific pathogenic molecules in the amoebae. However, it is known that epithelial cells exposed to pathogens respond in different ways to their presence. To investigate this, we measured damage of MDCK cells by *E. histolytica* trophozoites when the monolayers had been previously exposed to the enteropathogenic bacteria used in this study. We analyzed the interaction with amoebae that were not exposed to bacteria as well as with amoebae that had phagocytosed bacteria. As shown in the first set of bars in Figure 4A, damage to control monolayers by amoebae that phagocytosed bacteria was increased (compare *Eh*/MDCK with bars in the same set) corroborating results shown in Figure 1. The second set of bars shows that amoebae that had not ingested bacteria, but were co-cultured with epithelial cells exposed to bacteria, increased their cytopathic effect (compare values in this set of bars with those in the left). The third set of bars shows that amoebae that had phagocytosed bacteria, when co-cultured with monolayers exposed to pathogenic bacteria, greatly increased cell damage. In this case, there was not only more damage, but it occurred faster, as the monolayers were completely destroyed in 45 min by amoebae that had phagocytosed EPEC or *Shigella.*

The increased damage to monolayers exposed to bacteria suggested that the presence of bacteria could be inducing changes in the epithelial cells that facilitated adhesion of amoebae. Figure 4B shows in the first set of bars, the adhesion of amoebae that had phagocytosed bacteria to control MDCK cells. As also shown in Figure 2, these amoebae showed higher adhesion than amoebae that had not phagocytosed bacteria. The second set of bars shows that amoebae not incubated with bacteria adhered better if monolayers had been exposed to bacteria, especially *Shigella*. The third set of bars shows that after exposure of monolayers to bacteria, adhesion of amoebae that had phagocytosed bacteria reached the highest adhesion index, particularly after interaction with *Shigella.*

These results showed that MDCK cell monolayers exposed to bacteria, but particularly to pathogenic bacteria, are better targets for adhesion and damage by amoebae than unexposed monolayers. These two processes can be further enhanced if these monolayers were incubated with amoebae that had phagocytosed bacteria, in particular the pathogenic strains that, as shown above, also increased amoebic virulence.

### Release of chemotactic molecules and IL-8 in MDCK cells induced by pathogenic bacteria

**Figure 5:**
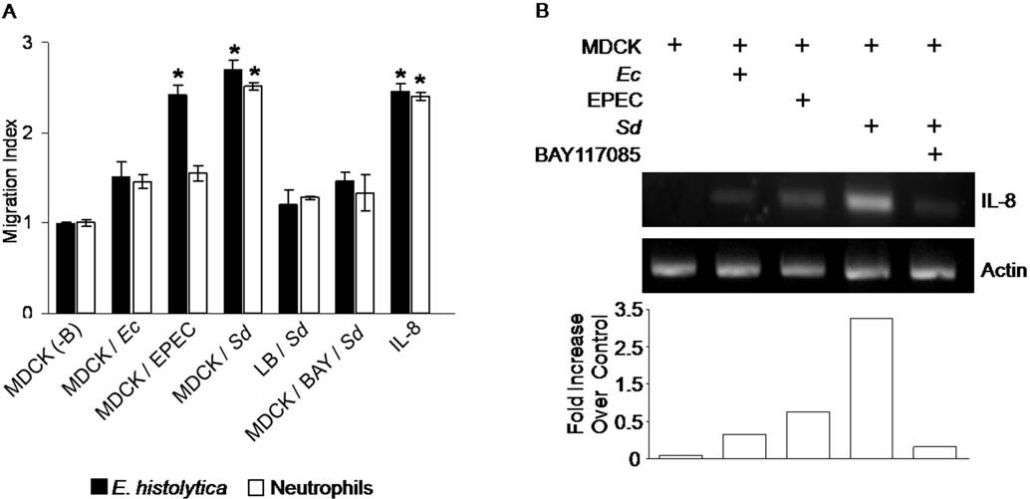
Release of chemotactic molecules and expression of IL-8 mRNA in MDCK cells induced by pathogenic bacteria. A, chemotaxis of *E. histolytica* and canine neutrophils to culture media from MDCK monolayers exposed to bacteria. Calcein-labeled amoebae and neutrophils were loaded on Transwell migration chambers containing in the lower chamber culture media from MDCK monolayers exposed to bacteria for 4 h. Chemotaxis to IL-8 (100 ng/ml) was determined by addition of the cytokine into the lower chambers. MDCK/BAY/*Sd* bars show migration to culture medium from cells exposed to *Shigella* in the presence of BAY11-7085. Bars LB/*Sd* indicate migration index to overnight culture medium of *Shigella*. The very low number of cells that migrated to control MDCK cell culture medium was given the index value = 1.0. Mean±SD, n = 6, * *p*≤0.05. B, a representative gel showing IL-8 mRNA expression induced in MDCK monolayers exposed to commensal *E.coli*, EPEC or *Shigella* for 4 h. Inhibition of IL-8 mRNA expression by BAY11-7085 was analyzed in monolayers incubated for 30 min before co-culture with *Shigella* and continuing the co-culture in the presence of the inhibitor. Expression of actin mRNA in the same MDCK cells was the loading control. Bars underneath show densitometric quantification of the bands in the gel where the significant increase of IL-8 expression induced by *Shigella* is shown.

Neutrophils and macrophages are not the only cells attracted by pro-inflammatory cytokines to participate in an inflammatory response of epithelia. *E. histolytica* trophozoites are also attracted to intestinal epithelial tissue when inflammatory cells are present [18],[34]. Recent reports have shown that *E. histolytica* trophozoites migrate in response to human TNFα and IL-1β [35],[36], suggesting a possible role for cytokines in the migration of amoebae to sites where bacteria are present and have initiated an inflammatory response. As shown in Figure 5A, amoebae and neutrophils were induced to migrate by culture media from MDCK cells exposed to bacteria. Culture media from MDCK monolayers exposed to *E. coli* induced a slight but not significant increase of migration of trophozoites and neutrophils, while culture media from EPEC *or Shigella*-exposed cells induced 2 times and almost 3 times higher migration of amoebae. Neutrophils were particularly attracted to culture medium from cells exposed to *Shigella.* Lack of response of amoebae and neutrophils to overnight medium of *Shigella* ruled out that migration could had been induced by bacterial products in the culture media. It has been reported that MDCK cells release IL-8 when subjected to *Salmonella typhimurium* infection [37]. The presence of IL-8 in the culture media of MDCK cells exposed to *Shigella* was corroborated by ELISA assays utilizing a monoclonal antibody to canine IL-8 (clone 258901, RD Systems Inc, Minneapolis, MN, kindly donated by Dr. A. Castillo, CINVESTAV). The results showed concentrations of the cytokine in three different culture media in the range of 180–200 pg/ml.

At the same time, it was found that human IL-8 induced migration of both neutrophils and amoebae, providing support to the chemoattractant role of this chemokine when present in culture media. Furthermore, we found that culture media from cells exposed to *Shigella* in the presence of the inhibitor of IL-8 mRNA expression, BAY11-7085 [38], reduced migration of both neutrophils and amoebae by 50%. If the inhibitor was added to culture media of cells after their exposure to *Shigella*, it had not effect on the migration of the amoebae (data not shown).

### MDCK cells exposed to bacteria express IL*-8* mRNA

It has been shown that epithelial cells in culture release pro-inflammatory cytokines, such as IL-8 as a defense mechanism when infected by bacteria [39],[40]. *Shigella* infection of intestinal cells activates NFκB through a polysaccharide-dependent innate intracellular response leading to the expression of this cytokine [39],[41]. Other enteropathogenic bacteria also activate release of cytokines [37],[39],[40],[41]. Changes in transcription patterns induced by pathogens could provide a clear indication of the response mechanisms of infected cells. Our data above showed that enterobacteria induced important changes in amoebae and epithelial cells. The changes induced by pathogenic bacteria and especially by *Shigella* were always more pronounced. Thus, it was clear that the presence of bacteria was affecting the interaction between amoebae and epithelial cells. We analyzed the expression of IL-8 mRNA in cells exposed to *E. coli* 086∶H18, EPEC and *Shigella* by RT-PCR assays using specific primers for MDCK cell IL-8 mRNA designed for this purpose. We thought that it was very interesting that IL-8 was expressed in cells exposed to bacteria as a defense response, but at the same time this chemokine was acting as chemoattractant for the amoebae. Figure 5B shows the results of a representative experiment (out of three) where after 4 h of exposure of MDCK cells to *E. coli* 086∶H18, EPEC or *Shigella*, the expression of IL-8mRNA was differentially induced in cells exposed to enteropathogenic bacteria. The highest expression corresponded to cells exposed to *Shigella.* The figure also shows that the expression of IL-8 mRNA was inhibited 93% in MDCK cells treated with 10 µM BAY11-7085 before exposure to *Shigella.* These results revealed that exposure of MDCK cells to bacteria, but particularly to invasive bacteria like *Shigella*, can induce a signaling process that activates NFκB pathways and expression of IL-8 mRNA.

### Enteropathogenic bacteria induce morphological and functional alterations of MDCK monolayers

We then analyzed if the induction of IL-8 and its release induced structural or functional damage to the bacteria-exposed cells and how this response might be modulated by the presence of another pathogen. Co-culture of pathogenic enterobacteria with epithelial cells is reported to induce alterations of epithelial organization [16],[42]. Reorganization of the actin cortical cytoskeleton is closely associated with altered permeability of epithelia and endothelia elicited by the infection [15],[37],[40],[43],[44].

**Figure 6:**
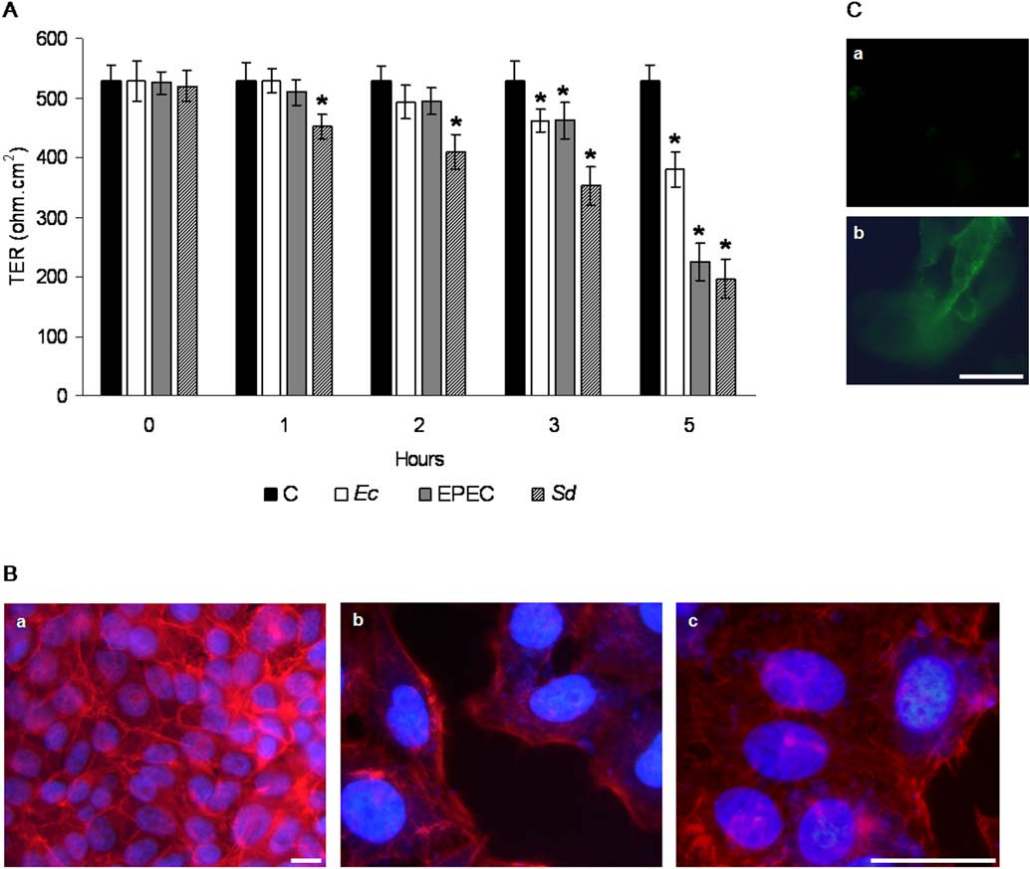
Enteropathogenic bacteria induce morphological and functional alterations of MDCK cell monolayers. A, TER was monitored in confluent monolayers at steady state (time 0) and at the indicated times after addition of bacteria. Bars labeled (C) show TER values in control monolayers not exposed to bacteria. The other bars show TER values in monolayers exposed to each one of the bacteria strains. Mean±SD, n = 9, * *p*≤0.05. B, Actin stained with Rhodamine-phalloidin in control monolayers not exposed to bacteria (a) and in monolayers exposed to EPEC (b) and *Shigella* (c). Bacteria and nuclei are stained with DAPI. Bar = 15 µm. C, FITC-Annexin V staining of MDCK cell monolayers, exposed to *Shigella* for 4 h (a) and after UV irradiation (b). Bar = 15 µm.

Signaling pathways and mechanisms activated by pathogens to disrupt the structural organization of target cells or to induce a cell response are relatively well known with pathogenic bacteria. In contrast, these aspects are only beginning to be explored in the case of pathogenic amoebae [45]. Thus, we determined if exposure of MDCK cells to enterobacteria modified structural and functional features of the MDCK monolayers that could explain increased amoebic damage. Figure 6A, shows that exposure of monolayers to all the bacteria strains caused gradual decrease of transepithelial resistance (TER), leading to higher permeability. The initial steady state TER values of approximately 528±33 ohm.cm^2^ dropped to 380±20 ohm.cm^2^ in monolayers exposed for 5 h to the commensal *E. coli* and to196±33 ohm.cm^2^ after exposure to EPEC or *Shigella.* Monolayers not exposed to bacteria maintained the initial steady state TER. Changes in the organization of the actin cytoskeleton, known to regulate opening of the tight junctions [30],[46], were assessed by staining cells exposed to bacteria with Rhodamine-phalloidin to visualize polymerized actin. As shown in Figure 6B, actin in control monolayers was forming juxtaposed cortical rings and fine actin filaments on the basolateral side of the cells and microvilli on the apical side, all of them characteristic features of confluent polarized MDCK monolayers (Figure 6B, a). In contrast, exposure to EPEC or *Shigella* (Figure 6B, b, c) induced separation of the cell borders, loss of the cortical actin ring and a striking reorganization of actin into thicker filaments. This rearrangement of actin filaments could be explained by bacteria-initiated disruption of the tight junction components and signaling to activate release of pro-inflammatory cytokines. These changes, not necessarily conducive to cell death (see Figure 6C, a, b) initiate the response to the presence of the parasite and its control by the release of pro-inflammatory cytokines.

## Discussion

When trophozoites of *Entamoeba histolytica* invade the host intestinal mucosa, they can cause inflammatory colitis. However, trophozoites can remain in the colon without causing tissue damage. Phagocytosis of bacteria, regularly present in the colonic flora, has been considered a possible stimulus to induce amoebic invasive behavior. In regions were *E. histolytica* and *E. dispar* are endemic, it is common that intestinal infections caused by enteropathogenic bacteria occur simultaneously with the presence of amoebae [21],[22],[23],[24],[25],[26]. In these conditions, it is also common to find exacerbated manifestations of the infection. It is then feasible that the interplay between pathogens modulates amoebic virulence and the response of the intestinal epithelial cells.

To approach this important aspect of mixed infections, so far poorly examined, we utilized an *in vitro* system where we could test virulence of *E. histolytica* and *E. dispar* trophozoites after phagocytosis of enteropathogenic bacteria and, at the same time, assess if interaction of enteropathogenic bacteria with epithelial cells elicited responses that could modify amoebic damage. The *E. coli*, commensal 086∶H18 strain, the pathogenic EPEC, as well as an invasive isolate of *S. dysenteriae* were chosen for this study. Although the natural habitat of EPEC is the not the colon, this non-invasive pathogen was readily phagocytosed by amoebae and it is often present in mixed intestinal infections and has been utilized for *in vitro* bacterial infection of MDCK monolayers [14],[30],[37],[47]. Additionally, MDCK monolayers are a well characterized epithelial system, known to retain structural, functional and molecular features of polarized epithelia.

A surprising first result was that only *E. histolytica* trophozoites digested the phagocytosed bacteria, although *Shigella* retained 70% viability for more than 12 h while, all the bacteria phagocytosed by *E. dispar* were fully viable after 24 h (Figure S1, Text S1). Survival mechanisms of *Salmonella* and *Shigella* to escape digestion in highly phagocytic cells are well characterized [47],[48],[49]. Although a similar situation may prevent digestion of *Shigella* by *E. histolytica*, no studies have been done respect to the mechanisms utilized by pathogenic bacteria to survive in *Entamoeba.* To our knowledge, this is the first report to address this interesting phenomenon, at the moment beyond the scope of this study.

In spite of their different digestive capacity, trophozoites of *E. histolytica* that had phagocytosed EPEC, and particularly *Shigella*, caused higher damage to MDCK cells. In contrast, *E. dispar*, under the same conditions, did not cause any noticeable cytopathic effect. *E. histolytica* trophozoites have on their surface a protein complex with cell adhesive properties, the Gal/GalNAc lectin [13],[50]. So, increased adhesion to cells could reflect higher levels of the lectin on the trophozoite surface. Indeed, higher levels of the lectin were found in amoebae that showed increased adherence and caused more damage to target cells. Competition experiments showed that although increased adhesion was related to increased lectin expression on the surface, binding was also due to other molecules on the amoebae. Competition of the binding by galactose, the main ligand of the lectin, supported this conclusion. Expression of cysteine proteinases CP2 and CP5 in trophozoites is also a factor in cell damage [7],[51],[52], although with exception of CP5, other major CPs are also expressed in the non-pathogenic *E. dispar* [6]. Analysis of cysteine proteinase activities in lysates and culture media of amoebae that phagocytosed bacteria showed a selective increase of some of the major activities, both in amoebic lysates and culture media. Therefore, one possibility could be that the increased cell damage inflicted by amoebae that phagocytosed EPEC and *Shigella* is due to higher CP activities released during increased adhesion to target cells. Specific inhibition of cysteine proteinases in co-cultures of amoebae and epithelial cells blocked the increase of cytopathic activities shown by amoebae after ingestion of pathogenic bacteria. In contrast, no significant increases in CP activities were found in *E. dispar* which could not digest phagocytosed bacteria (data not shown). These data are very suggestive of an induction of the activity of these proteins after phagocytosis and digestion of enteropathogenic bacteria. To this moment, analyses of amoebic microarrays and phagosomes have not provided any clues for the presence of molecules that could explain the increase in amoebic virulence and the survival of *Shigella* in the *E. histolytica* [47],[53].

It has been shown that pro-inflammatory cytokines are released by intestinal cells exposed to *E. histolytica* infection [18] and that recombinant amoebic proteinases are capable of cleaving pro-inflammatory cytokine precursors to their active form *in vitro* [34]. It is possible then that increased adhesion of amoebae to epithelial cells together with higher release of CP into the medium, induced by phagocytosis of bacteria and particularly by pathogenic bacteria, would lead to higher concentrations of active cytokines at the sites of contact between amoebae and epithelial cells. The presence of activated inflammatory cytokines would then attract neutrophils and other cells to the sites where amoebae concentrate.

Our *in vitro* model allowed testing the above mentioned possibilities. It has been shown that several pathogens increase permeability of epithelial cells. *E. coli* ETEC and EPEC strains diminish the barrier functions of cultured epithelial monolayers [16],[17]. *Salmonella* and *Shigella* disrupt the intercellular junctions by alteration of the normal distribution of molecules that associate with them and disrupt the organization of the cytoskeleton [37],[43]. Virus entry into cells also results in this type of cellular disruption [44]. Alteration of epithelial barriers allows penetration of pathogens into the paracellular space and their dissemination into lower cell layers, as well as migration of inflammatory cells to the luminal side [14],[16],[17],[47],[54]. *E. histolytica* trophozoites can also produce decrease of TER in cultured epithelial cells [45].

We tested the effect of enterobacteria on monolayer permeability. TER registers indicated a small decrease in monolayers exposed to the commensal *E. coli*, and a gradual, but more accentuated drop in monolayers exposed to EPEC or *Shigella.* After 5 h, TER values had decreased to levels indicative of complete opening of the intercellular junctions. Unsealed monolayers allow passage not only of ions and big molecules, but even of neutrophils and other cells of the immune system. Opening of the tight junctions also allows exposure of receptors for these cells and for pathogens [47]. For example, *H. pylori* and *L. monocytogenes* have proteins that bind to E-cadherin once the tight junctions of the epithelial cells are opened, so they can enter cells or epithelial layers [14],[55]. We found that trophozoites increased their adhesion to MDCK cells that had been exposed to bacteria, ie: with their membrane junctions unsealed. Adhesion was even higher if trophozoites had phagocytosed bacteria. Higher adhesion could be related to the higher cell damage by trophozoites observed in epithelial cells exposed to bacteria, in particular to EPEC or *Shigella.*

These results corroborate that an increase of adhesion to cells by amoebae results in more cell damage. The increase was induced in trophozoites after phagocytosis of bacteria or after exposure of epithelial cells to the same bacteria. Moreover, the highest adherence and cell damage were observed when both, trophozoites and epithelial cells were incubated with bacteria. As shown above, phagocytosis of bacteria induced higher levels of the Gal/GalNAc lectin on the trophozoite's surface, which would facilitate adhesion of the amoebae. However, this would require higher number of receptors on the target cells for a better interaction. Our data suggest that other molecules on the amoebic surface could be participating in the interaction. Preliminary results, currently investigated in our laboratory, have shown that amoebae can induce exposure of TLRs on the surface of intestinal epithelial cells.

The gradual drop of TER in monolayers exposed to EPEC or *Shigella*, suggests a gradual effect on the disorganization of the intercellular junctions that was not apoptotic, but capable of inducing a marked reorganization of the actin cytoskeleton. Disruption of the cortical actin circumferential ring, loss of microvilli and rearrangement of the basolateral filaments, have been correlated with the opening of the tight junctions [30],[46], normally a reversible process that allows epithelial cells to adapt to conditions in the medium. However, disorganization of intercellular junctions by enteropathogenic bacteria and other pathogens leads to release of pro-inflammatory cytokines [17],[18],[19],[40]. Our data have shown that MDCK epithelial cells co-cultured with enteropathogenic bacteria suffered functional alterations and released, into the medium, molecules capable of activating chemotaxis of amoebae and neutrophils. Both types of cells were specifically attracted to pro-inflammatory cytokine IL-8. IL-8 is released by epithelial cells during the interaction with pathogens and acts as a chemokine, playing an important role in the migration of neutrophils to sites of infection [37],[39],[40]. We found that MDCK cells exposed to bacteria induced expression of IL-8 mRNA and release of this chemokine to the culture media, corroborating previous results by other authors [39],[40],[41],[44]. Induction was low after exposure to the commensal *E. coli*, but increased markedly after exposure to *Shigella.* The induction was almost completely inhibited by inactivation of NFκB. Although this transcription factor activates transcription of different genes [39], the fact that IL-8 mRNA expression could be differentially induced by the exposure of MDCK cells to different bacteria, and was blocked by the inhibitor of NFκB activation, strongly suggest that bacteria, and particularly *Shigella*, can activate signaling pathways leading to expression of IL-8 mRNA [15],[39],[40],[47]. We showed here, that canine neutrophils and amoeba migrated in a similar way to human IL-8 and to culture media of MDCK cells previously exposed to bacteria. The highest migration was registered for the culture media from MDCK cells exposed to *Shigella* where the presence of IL-8 was corroborated by ELISA. Moreover, these cells showed the highest induction of IL-8 mRNA. Previous experiments from our group have shown that amoebae also respond to IL-1β [36] and recently, it has been reported that *E. histolytica* trophozoites respond to human TNFα gradients by chemotactic sliding [35]. The chemotactic effect of inflammatory cytokines on amoebae supports the idea that amoebae can reach sites in the epithelia where an inflammatory response has been started by bacteria. At the same time, amoebae present in the same milieu can increase their virulence by phagocytosis of bacteria and cells that have been altered by the presence of bacteria are more susceptible to adherence and damage by amoebae. It is possible that all of these phenomena contribute to the pathogen's ability to penetrate epithelial layers. What could be the role of *E. dispar* in this situation? The avirulence of *E. dispar* suggests a non-aggressive participation of this amoeba in mixed infections. However, its ubiquitous presence in samples from patients, its inability to digest ingested bacteria (Text S1) and isolated reports of lesions produced by some amoebic isolates, make study of this amoeba species also worth pursuing.

A limitation of our *in vitro* cell system is the fact that we are observing phenomena outside of the intestine. However, with this approach to mixed amoeba/bacteria infections we have obtained results that could not have been monitored *in vivo.* We have now a better insight into the role played by the participating elements in the organism. A molecular approach to understand better the signaling processes and the molecules involved at different stages of the infection is now feasible. We hope that our findings encourage research on a health problem still prevalent and neglected in developing countries.

## Supporting Information

Figure S1 Viability of bacteria phagocytosed by amoebae. A, CFU of bacteria recovered from *E. histolytica*. B, CFU of bacteria recovered from *E. dispar*. After 2 h interaction of amoebae with bacteria, non-phagocyted bacteria were removed and amoebae cultured at the indicated times. After lysis of amoebae, recovered bacteria were allowed to grow for 24 h in LB plates before colony numbers were quantified. Mean±SD, n = 4, * *p*≤0.05.
(0.66 MB TIF)

Figure S2 Silver-stained representative gels of lysates and culture media from amoebae after phagocytosis of bacteria. 2 µg/ml of protein were loaded per lane in the case of lysates and 5 µl/lane in the case of culture medium.
(0.63 MB TIF)

Text S1 Additional Material
(0.03 MB DOC)

Alternative Language Abstract S1 Translation of the Abstract into Spanish by Isaura Meza
(0.03 MB DOC)

## References

[1] Espinosa-Cantellano M, Martínez-Palomo A (2000). Pathogenesis of intestinal amoebiasis: from molecules to disease.. Clin Microbiol Rev.

[2] Stanley SL (2001). Pathophysiology of amoebiasis.. Trends Parasitol.

[3] Bruchhaus I, Jacobs T, Leippe M, Tannich E (1996). *Entamoeba histolytica* and *Entamoeba dispar*: differences in numbers and expression of cysteine proteinase genes.. Mol Microbiol.

[4] Espinosa-Cantellano MA, González-Robles A, Chávez B, Castañón G, Argûello C (1998). *Entamoeba dispar*: Ultrastructure, surface properties, and cytopathic effect.. J Eukaryot Microbiol.

[5] Zaki M, Meelup P, Sun W, Clark CG (2002). Simultaneous differentiation and typing of *Entamoeba histolytica* and *Entamoeba dispar*.. J Clin Microbiol.

[6] Bruchhaus I, Loftus BJ, Hall N, Tannich E (2003). The intestinal protozoan parasite *Entamoeba histolytica* contains 20 cysteine protease genes, of which only a small subset is expressed during in vitro cultivation.. Eukaryot Cell.

[7] Hellberg A, Nickel R, Lotter H, Tannich E, Bruchhaus I (2001). Overexpression of cysteine proteinase 2 in *Entamoeba histolytica* or *Entamoeba dispar* increases amoeba-induced monolayer destruction in vitro but does not augment amoebic liver abscess formation in gerbils.. Cell Microbiol.

[8] MacFarlane R, Singh U (2006). Identification of differentially expressed genes in virulent and nonvirulent *Entamoeba* species: Potential implications for amoebic pathogenesis.. Infect Immun.

[9] Blessmann J, Ali IK, Nu PA, Dinh BT, Viet TQ (2003). Longitudinal study of intestinal *Entamoeba histolytica* infections in asymptomatic adult carriers.. J Clin Microbiol.

[10] Ramos F, Mora P, González E, García G, Ramiro M (2005). High prevalence rate of *Entamoeba histolytica* asymptomatic infection in a rural mexican community.. Am J Trop Med Hyg.

[11] Bracha R, Kobiler D, Mirelman D (1982). Attachment and ingestion of bacteria by trophozoites of *Entamoeba histolytica*.. Infec Immun.

[12] Mirelman D (1987). Amoeba-bacterium relationship in amoebiasis.. Microbiol Rev.

[13] Padilla-Vaca F, Ankri S, Bracha R, Koole L, Mirelman D (1999). Down-regulation of *Entamoeba histolytica* virulence by monoxenic cultivation with *Escherichia coli* O55 is related to a decrease in expression of the light (35-kilodaltons) subunit of the Gal/Gal-NAc lectin.. Infect Immun.

[14] Bagnoli F, Buti L, Tompkins L, Covacci A, Amieva MB (2005). *H. pylori* Cag A induces transition from polarized to invasive phenotype in MDCK cells.. Proc Natl Acad Sci USA.

[15] Bruewer M, Hopkins AM, Hobert ME, Nusrat A, Madara JL (2004). Rho A, Rac1 and Cdc42 exert distinct effects on epithelial barrier via selective structural and biochemical modulation of junctional proteins and F-actin.. Am J Physiol Cell Physiol.

[16] Canil LC, Rosenshine I, Ruschkowski S, Donnenberg MS, Kaper JB (1993). Enteropathogenic *E. coli* decreases the transepithelial electrical resistance of polarizad epithelial monolayers.. Infec Immun.

[17] Spitz J, Yuhan R, Koutsouris A, Blatt C, Alverdy J (1995). Enteropathogenic *Escherichia coli* adherence to intestinal epithelial monolayers diminishes barrier function.. Am J Physiol.

[18] Seydel KB, Li E, Swanson PE, Stanley SL (1997). Human intestinal epithelial cells produce pro-inflammatory cytokines in response to infection in SCID mouse-human intestinal xenograph model of amoebiasis.. Infect Immun.

[19] Seydel KB, Li E, Zhang Z, Stanley SL (1998). Epithelial cell-initiated inflammation plays a crucial role in early tissue damage in amoebic infection of human intestine.. Gastroenterol.

[20] Zhang Z, Jin L, Champion G, Seydel KB, Stanley SL (2001). *Shigella* infection in SCID mouse-human intestinal xenograft model: role for neutrophils in containing bacterial dissemination in human intestine.. Infect Immun.

[21] Chatterjee BD, Thawani G, Sanyal SN (1989). Etiology of acute childhood diarrhea in Calcutta.. Trop Gastroenterol.

[22] Flores-Abuxapqui JJ, Suárez –Hoil GJ, Puc-Franco MA, Heredia-Navarrete MR, Franco-Monsreal J (1993). Prevalence of enteropathogens in children with liquid diarrhea.. Rev Latinoamer Microbiol.

[23] Gatti S, Swierczynski J, Cevini C, Bruno A, Anselmi M (2000). Incidence of amoebic infection in a village of northern Ecuador.. Arch Med Res.

[24] Haque R, Mondal D, Kirkpatrick BD, Akther S, Farr BM (2003). Epidemiologic and clinical characteristics of acute diarrhea with emphasis on *Entamoeba histolytica* infections in preschool children in an urban slum of Dhaka, Bangladesh.. Am J Trop Med Hyg.

[25] Lara R, Galindo E, Olarte J, Hernández G (1974). Mixed infections by *Entamoeba histolytica*, *Shigella* and other enteropathogenic bacteria found in children with diarrhea.. Arch Invest Med (Mex).

[26] Orlandi PP, Silva T, Magalhaes GF, Alves F, de Almeida Cunha RP (2001). Enteropathogens associated with diarrheal disease in infants of poor urban areas of Porto Velho, Rondonia: a preliminary study.. Mem Inst Oswaldo Cruz.

[27] Ramos F, Valdez E, Morán P, González E, Padilla G (2000). Prevalence of *Entamoeba histolytica* and *Entamoeba dispar* in a highly endemic rural population.. Arch Med Res.

[28] Diamond LS, Harlow DR, Cunnick CC (1978). A new medium for axenic cultivation of *Entamoeba histolytica* and other *Entamoeba*.. Trans R Soc Trop Med Hyg.

[29] Clark CG, Diamond LD (2002). Methods for cultivation of luminal parasitic protists of clinical importance.. Clin Microbiol Rev.

[30] Castillo AM, Reyes JL, Sánchez E, Mondragón R, Meza I (2002). 2,3-Butanedione monoxime (BDM), a potent inhibitor of myosin-actin interaction, induces ion and fluid transport in MDCK monolayers.. J Mus Res Cell Motil.

[31] Galván-Moroyoqui JM (2006). Interacción de *Entamoeba histolytica* y *Entamoeba dispar* con bacterias enteropatógenas.. MSc thesis, Department of Molecular Biomedicina, CINVESTAV-IPN, México.

[32] Franco E, de Araujo-Soares RM, Meza I (1999). Specific and reversible inhibition of *Entamoeba histolytica* cysteine-proteinase activities by Zn^2+^: implications for adhesion and cell damage.. Arch Med Res.

[33] Franco-Barraza J, Zamudio-Meza H, Franco E, Domínguez-Robles MC, Villegas-Sepúlveda N (2006). Rho-signaling in *Entamoeba histolytica* modulates actomyosin-dependent activities stimulated during invasive behavior.. Cell Motil Cytoskeleton.

[34] Zhang Z, Yan L, Wang L, Seydel KB, Li E (2000). *Entamoeba histolytica* cysteine proteinases with interleukin-1 beta converting enzyme (ICE) activity cause intestinal inflammation and tissue damage in amoebiasis.. Mol Microbiol.

[35] Blázquez S, Zimmer C, Guigon G, Olivo-Marín JC, Guillén N (2006). Human tumor necrosis factor is a chemoattractant for the parasite *Entamoeba histolytica.*. Infect Immun.

[36] Meza I, Galvàn-Moroyoqui M, Dominguez-Robles MC, Franco E (2007). *Entamoeba histolytica* and *E. dispar* interaction with enteropathogenic bacteria synergizes damage to epitheal cells amplifying the inflammatory response.. Eur J Trop Med Inter Health.

[37] Hobert ME, Sands KA, Mrsny RJ, Madara J (2001). Cdc42 and Rac1 regulate late events in *Salmonella typhimurium* induced interleukin-8 secretion from polarized epithelial cells.. J Biol Chem.

[38] Pierce JW, Schoenleber R, Jesmok G, Moore SA, Collins T (1997). Novel inhibitors of cytokine-induced Iκ, Bα phosphorylation and endothelial cell adhesion molecule expression show anti-inflammatory effects in vivo.. J Biol Cem.

[39] Philpott DJ, Yamaoka S, Israel A, Sansonetti PJ (2000). Invasive *Shigella flexneri* activates NFκB through an LPS-dependent innate intracellular response and leads to IL-8 expression in epithelial cells.. J. Immunol.

[40] Steiner TS, Lima AA, Nataro JP, Guerrant RL (1998). Enteroaggregative *Escherichia coli* produce intestinal inflammation and growth impairment and cause interleukin-8 release from intestinal cells.. J Infect Dis.

[41] Singer M, Sansonetti PJ (2004). IL-8 is a key chemokine regulating neutrophil recruitment in a new mouse model of *Shigella*-induced colitis.. J Immunol.

[42] Pentecost M, Otto G, Theriot JA, Amieva MR (2006). *Listeria monocytogenes* invades epithelial junctions at sites of cell extrusion.. PLoS Pathol.

[43] Finlay BB, Ruschkowski S, Dedhar S (1991). Cytoskeletal arrangements accompanying *Salmonella* entry into epithelial cells.. J Cell Sci.

[44] Talavera D, Castillo AM, Domínguez-Robles MC, Escobar-Gutiérrez A, Meza I (2004). IL-8 release, tight junction and cytoskeleton dynamic reorganization conducive to permeability increase are induced by dengue virus infection of microvascular endothelial monolayers.. J Gen Virol.

[45] Leroy A, Lauwaet T, De Bruyne G, Cornelissen M, Marrel M (2000). *Entamoeba histolytica* disturbs the tight junction complex in human enteric T84 cell layers.. FASEB J.

[46] Meza I, Ibarra G, Sabanero M, Martinez-Palomo A, Cereijido M (1980). Occluding junctions and cytoskeletal components in a cultured transporting epithelium.. J Cell Biol.

[47] Phalipon A, Sansonnetti PJ (2007). *Shigella's* ways of manipulating the host intestinal innate and adaptive immune system: a tool box for survival.. Immunol Cell Biol.

[48] Rosales-Reyes R, Alpuche-Aranda C, Ramírez-Aguilar ML, Castro-Eguiluz AD, Ortiz-Navarrete V (2005). Survival of *Salmonella enterica* serovar *Typhimurium* within late endosomal-lysosomal compartments of B lymphocytes is associated with the inability to use the vacuolar alternative major histocompatibility complex class I antigen-processing pathway.. Infect Immun.

[49] Salcedo S, Cohen NS, Holden N (2001). Intracellular replication of *Salmonella typhimurium* strains in specific subsets of splenic macrophages in vivo.. Cell Microbiol.

[50] Petri WA, Mann BJ (1993). Molecular mechanisms of invasion by *Entamoeba histolytica*.. Semin Cell Biol.

[51] Mirelman D, Anbar M, Nuchamowitz Y, Bracha R (2006). Epigenetic silencing of gene expression in *Entamoeba histolytica*.. Arch Med Res.

[52] Tillack M, Nowak N, Lotter H, Bracha R, Mirelman D (2006). Increased expression of the major cysteine proteinases by stable episomal transfection underlines the important role of EhCP5 for the pathogenicity of *Entamoeba histolytica*.. Mol Biochem Parasitol.

[53] Foubister V, Rosenshine I, Donnenberg S, Finlay BB (1994). The *cacB* gene of enteropathogenic *E. coli* (EPEC) is necessary for signal transduction in epithelial cells.. Infect Immun.

[54] Sakaguchi T, Kohler H, Gu X, Mc Cormick BA, Reinecker HC (2002). *Shigella flexneri* regulates tight junction-associated proteins in human intestinal epithelial cells.. Cell Microbiol.

[55] Mengaud J, Ohayon H, Gounon P, Mege RM, Cossart P (1996). E-cadherin is the receptor for internalin, a surface protein required for entry of *L monocytogenes* into epithelial cells.. Cell.

