# Supplementary figures and images for: The Interplay between Entamoeba and Enteropathogenic Bacteria Modulates Epithelial Cell Damage
Source: PLoS Negl Trop Dis. 2008 Jul 23;2(7):e266. doi: 10.1371/journal.pntd.0000266 (PMC2447883; doi:10.1371/journal.pntd.0000266)

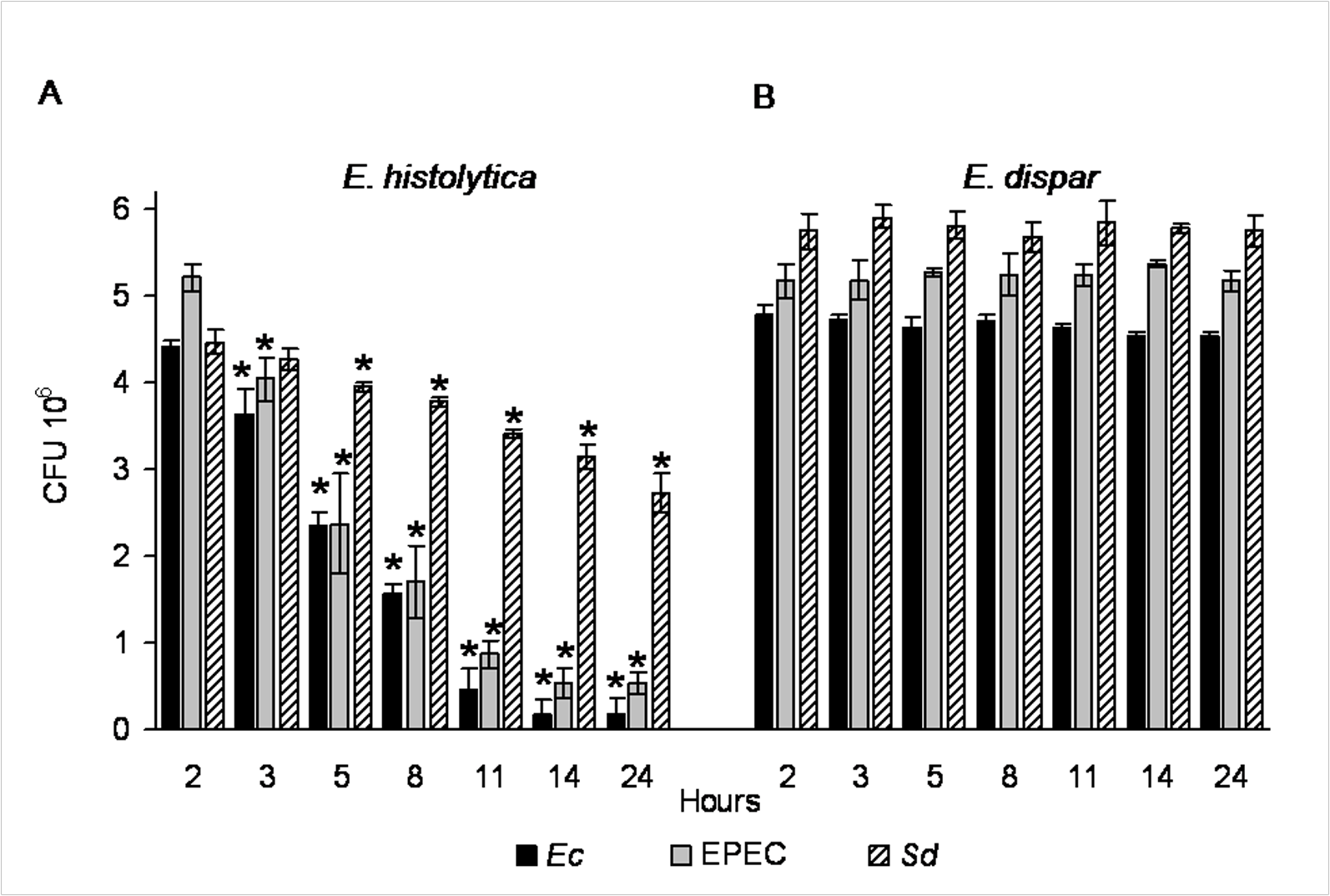

Supplement: Figure S1 — Viability of bacteria phagocytosed by amoebae. A, CFU of bacteria recovered from E. histolytica. B, CFU of bacteria recovered from E. dispar. After 2 h interaction of amoebae with bacteria, non-phagocyted bacteria were removed and amoebae cultured at the indicated times. After lysis of amoebae, recovered bacteria were allowed to grow for 24 h in LB plates before colony numbers were quantified. Mean±SD, n = 4, * p≤0.05. (0.66 MB TIF) [file pntd.0000266.s001.tif]

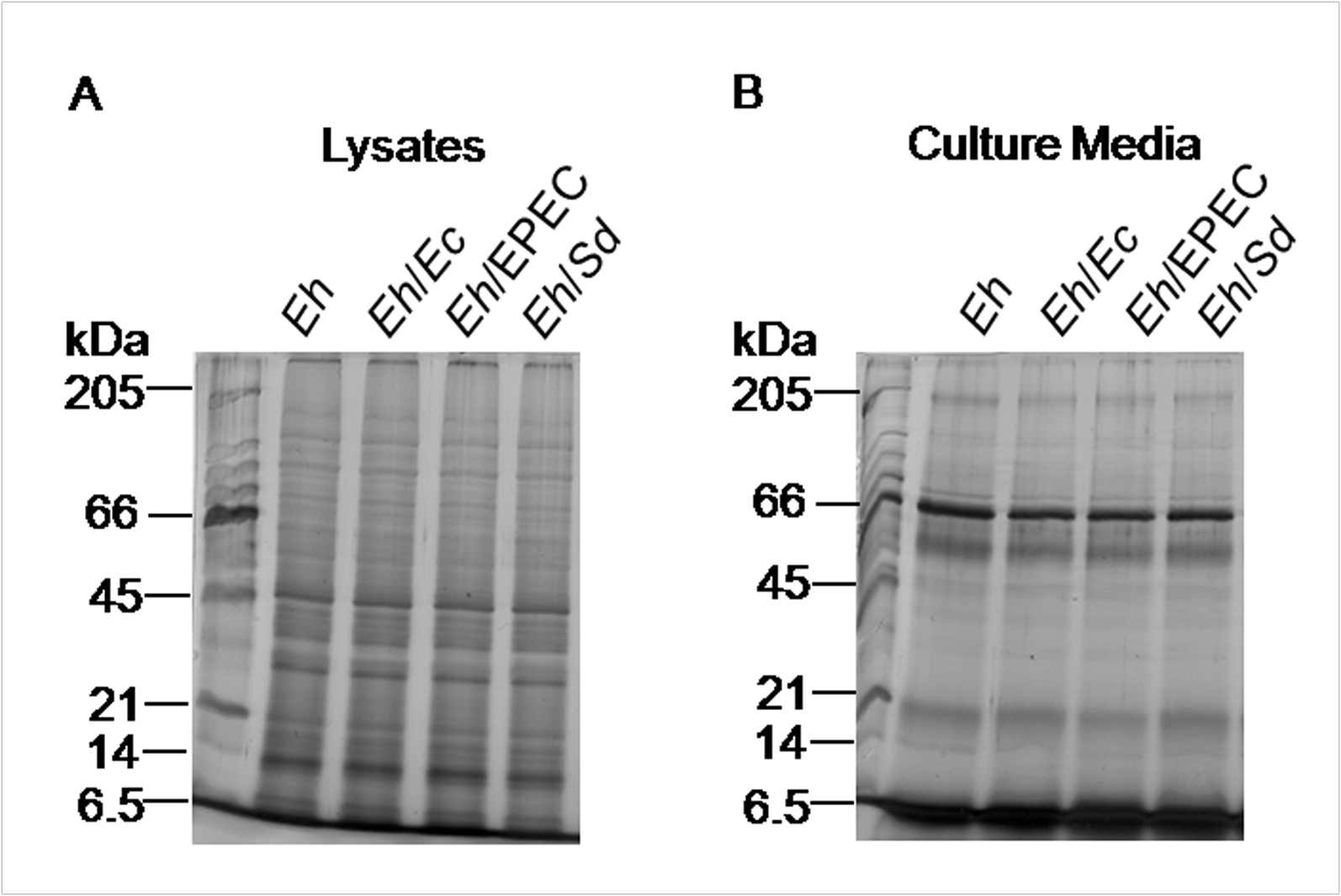

Supplement: Figure S2 — Silver-stained representative gels of lysates and culture media from amoebae after phagocytosis of bacteria. 2 µg/ml of protein were loaded per lane in the case of lysates and 5 µl/lane in the case of culture medium. (0.63 MB TIF) [file pntd.0000266.s002.tif]
