# Supplementary material for: The Interplay between Entamoeba and Enteropathogenic Bacteria Modulates Epithelial Cell Damage
Source: PLoS Negl Trop Dis. 2008 Jul 23;2(7):e266. doi: 10.1371/journal.pntd.0000266 (PMC2447883; doi:10.1371/journal.pntd.0000266)
Supplement: Text S1 — Additional Material (0.03 MB DOC) [file pntd.0000266.s003.doc]

**ADDITIONAL MATERIAL**

**Viability of bacteria phagocytosed by amoebae.**  The data in Figure 1 showed that phagocytosis of any of the strains of bacteria did not induce virulence in *E. dispar.* .As this non-pathogenic *Entamoeba* species has been reported in many cases of mixed intestinal infections, it was included in the study and we think it is important to valorate its possible contribution. Assessing viability of phagocytosed bacteria in *E. dispar*, we found that all the bacteria strains survived for long periods of time and were capable to form colonies in culture after their recovery from amoebae. In contrast, bacteria phagocytosed by *E. histolytica* were rapidly digested with exception of *Shigella* which retained 70 % viability for at least 24 h after been phagocytosed (Fig 1 Additional). This observation, reported here for the first time, called our attention in the context of survival mechanism of pathogens in highly phagocytic cells. *Acanthamoeba* and the amoebic form of *Dictyostelium* are nowadays been intensely studied as vectors of infective bacteria (1,2). The survival of bacteria in *Entamoeba* could be an important factor to consider in the milieu of an intestinal mixed infection. The results of bacteria survival in amoebae are presented as additional material.

REFERENCES

1. Unal C, Steinert M. 2006. *Dictyostelium discoideum* as a model to syudy host-pathogen interactions. Methods Mol Biol 346: 507-15.
2. Skriwan C, Fajardo M, Hagele S, Horn M, Wagner M, Michel R, Kronhe G, Schleicher M, Hacker J, Steinert M. 2002. Various bacterial pathogens and simbionts infect the amoeba *Dictyostelium discoideum.* Int J Med Microbiol 291: 615-24.

**ADDITIONAL FIGURES**

FIG. 1. Viability of bacteria phagocytosed by amoebae. A, CFU of bacteria recovered from *E. histolytica.* B, CFU of bacteria recovered from *E. dispar*. After 2 h interaction of amoebae with bacteria, non-phagocyted bacteria were removed and amoebae cultured at the indicated times. After lysis of amoebae, recovered bacteria were allowed to grow for 24 h in LB plates before colony numbers were quantified. Mean ± SD, n = 4, * *p* ≤ 0.05.

FIG. 2. Silver-stained representative gels of lysates and culture media from amoebae after phagocytosis of bacteria. 2 µg/ml of protein were loaded per lane in the case of lysates and 5µl/lane in the case of culture medium.

**TRANSLATION OF THE ABSTRACT FROM ARTICLE “ THE INTERPLAY BETWEEN *ENTAMOEBA* AND ENTEROPATHOGENIC BACTERIA MODULATES EPITHELIAL CELL DAMAGE” INTO SPANISH BY ISAURA MEZA.**

Resumen

*Antecedentes*: Las infecciones intestinales mixtas con *E.histolytica/E.dispar/*Bacterias, en donde se encuentra exacerbación de la enfermedad, son comunes en regiones endémicas de Amibiasis. A pesar de esto, la interacción amiba-bacterias ha sido poco estudiada. *Metodologìa:* Trofozoitos de *E. .histoytica* y de *E. dispar* se cultivaron con bacterias enteropatógenas de las cepas EPEC y *Shigella dysenteriae* y una cepa comensal de *E .coli*. Las amibas que fagocitaron bacterias fueron analizadas para determinar su capacidad de producir daño citopático a monocapas de células epiteliales. La actividad de las cisteín-proteasas, la adhesión a células epiteliales y la concentración de lectina Gal/GalNAC en la superficie amibiana fueron analizadas en amibas que mostraron un aumento en virulencia. Cambios estructurales y funcionales, así como la inducción en la expresión de la citosina IL-8, se determinaron en monocapas epiteliales antes y después de haber sido expuestas a las bacterias. La quimiotaxis de amibas y neutrófilos hacia IL-8 humana y hacia medios de cultivo condicionados de células epiteliales expuestas a bacterias fue cuantificada. *Hallazgos Principales*: *E. histolytica* digirió rapidamente a las bacterias fagocitadas aunque en el caso de *S. dysenteriae,* las bacterias permanecieron viables en un 70% después haber sido ingeridas. La fagocitosis de bacterias patógenas incrementó el daño citopático causado por las amibas, la concentración de la lectina Gal/GalNac en la superficie de las amibas y la actividad de las cisteín-proteasas. *E. dispar* en cambio se mantuvo avirulenta. La adhesión de las amibas y el daño a las células expuestas a bacterias se incrementaron. Incrementos adicionales fueron observados si las amibas habían fagocitado bacterias. Co-cultivo de las células epiteliales con bacterias enteropatógenas alteraron la permeabilidad de la monocapa epitelial e indujeron la expresión de IL-8. Medios de estos co-cultivos y la IL-8 recombinante humana mostraron capacidad quimioatrayente para los neutrófilos y para *E.histolytica*. *Conclusiones*: Monocapas de células epiteliales expuestas a bacterias enteropatógenas son más susceptibles al daño por *E.histolytica*. Al mismo tiempo, la fagocitosis de bacterias patógenas por las amibas aumenta aún más el daño a las células epiteliales. *Relevancia:* El sistema *in vitro* implementado permitió obtener datos que muestran que la interacción *Entamoeba* /bacterias enteropatògenas modula la respuesta de las células epiteliales hacia los patógenos. En infecciones intestinales mixtas, en donde tales interacciones son posibles, éstas podrían regular las manifestaciones de la enfermedad. Estos resultados proporcionan una visión novedosa para continuar investigando sobre este fenómeno.
