# Supplementary material for: The Interplay between Entamoeba and Enteropathogenic Bacteria Modulates Epithelial Cell Damage
Source: PLoS Negl Trop Dis. 2008 Jul 23;2(7):e266. doi: 10.1371/journal.pntd.0000266 (PMC2447883; doi:10.1371/journal.pntd.0000266)
Supplement: Alternative Language Abstract S1 — Translation of the Abstract into Spanish by Isaura Meza (0.03 MB DOC) [file pntd.0000266.s004.doc]

Resumen

*Antecedentes*: Las infecciones intestinales mixtas con *E.histolytica/E.dispar/*Bacterias, en donde se encuentra exacerbación de la enfermedad, son comunes en regiones endémicas de Amibiasis. A pesar de esto, la interacción amiba-bacterias ha sido poco estudiada. *Metodologìa:* Trofozoitos de *E. .histoytica* y de *E. dispar* se cultivaron con bacterias enteropatógenas de las cepas EPEC y *Shigella dysenteriae* y una cepa comensal de *E .coli*. Las amibas que fagocitaron bacterias fueron analizadas para determinar su capacidad de producir daño citopático a monocapas de células epiteliales. La actividad de las cisteín-proteasas, la adhesión a células epiteliales y la concentración de lectina Gal/GalNAC en la superficie amibiana fueron analizadas en amibas que mostraron un aumento en virulencia. Cambios estructurales y funcionales, así como la inducción en la expresión de la citosina IL-8, se determinaron en monocapas epiteliales antes y después de haber sido expuestas a las bacterias. La quimiotaxis de amibas y neutrófilos hacia IL-8 humana y hacia medios de cultivo condicionados de células epiteliales expuestas a bacterias fue cuantificada. *Hallazgos Principales*: *E. histolytica* digirió rapidamente a las bacterias fagocitadas aunque en el caso de *S. dysenteriae,* las bacterias permanecieron viables en un 70% después haber sido ingeridas. La fagocitosis de bacterias patógenas incrementó el daño citopático causado por las amibas, la concentración de la lectina Gal/GalNac en la superficie de las amibas y la actividad de las cisteín-proteasas. *E. dispar* en cambio se mantuvo avirulenta. La adhesión de las amibas y el daño a las células expuestas a bacterias se incrementaron. Incrementos adicionales fueron observados si las amibas habían fagocitado bacterias. Co-cultivo de las células epiteliales con bacterias enteropatógenas alteraron la permeabilidad de la monocapa epitelial e indujeron la expresión de IL-8. Medios de estos co-cultivos y la IL-8 recombinante humana mostraron capacidad quimioatrayente para los neutrófilos y para *E.histolytica*. *Conclusiones*: Monocapas de células epiteliales expuestas a bacterias enteropatógenas son más susceptibles al daño por *E.histolytica*. Al mismo tiempo, la fagocitosis de bacterias patógenas por las amibas aumenta aún más el daño a las células epiteliales. *Relevancia:* El sistema *in vitro* implementado permitió obtener datos que muestran que la interacción *Entamoeba* /bacterias enteropatògenas modula la respuesta de las células epiteliales hacia los patógenos. En infecciones intestinales mixtas, en donde tales interacciones son posibles, éstas podrían regular las manifestaciones de la enfermedad. Estos resultados proporcionan una visión novedosa para continuar investigando sobre este fenómeno.
